# Curcumin inhibits ovarian cancer progression by regulating circ-PLEKHM3/miR-320a/SMG1 axis

**DOI:** 10.1186/s13048-021-00916-8

**Published:** 2021-11-16

**Authors:** Sifan Sun, Hailiang Fang

**Affiliations:** 1 Department of Rehabilitation, The Second Hospital of Dalian Medical University, Dalian City, Liaoning Province China; 2 Department of Traditional Chinese Medicine, The Second Hospital of Dalian Medical University, No.467 Zhongshan Road, Shahekou District, Dalian, 116023 Liaoning Province China

**Keywords:** Ovarian cancer, Curcumin, Circ-PLEKHM3, miR-320a, SMG1

## Abstract

**Background:**

Curcumin has a potential therapeutic role in ovarian cancer. However, whether curcumin plays anti-cancer role in ovarian cancer by mediating the circular RNA (circRNA)/microRNA (miRNA)/mRNA network is still unclear.

**Methods:**

The expression of circ-PLEKHM3, miR-320a, and suppressor of morphogenesis in genitalia 1 (SMG1) was detected via qRT-PCR. Cell viability, colony-formation ability and apoptosis were analyzed via cell counting kit-8 assay, colony formation analysis, and flow cytometry. Protein expression was measured using western blot. The in vivo experiments were performed using a xenograft model. Target association was evaluated via dual-luciferase reporter analysis and RIP assay.

**Results:**

Curcumin suppressed ovarian cancer cell proliferation and promoted apoptosis. Circ-PLEKHM3 was downregulated in ovarian cancer, and its expression could be promoted by curcumin treatment. Circ-PLEKHM3 overexpression exacerbated the effect of curcumin on ovarian cancer cell proliferation and apoptosis, as well as anti-tumor effect. MiR-320a was targeted by circ-PLEKHM3. The inhibition effect of circ-PLEKHM3 overexpression on cell proliferation and the enhancing effect on cell apoptosis could be reversed by miR-320a mimic. SMG1 was targeted by miR-320a, and its knockdown also reversed the regulation of miR-320a inhibitor on the proliferation and apoptosis of ovarian cancer cells. In addition, circ-PLEKHM3 could upregulate SMG1 expression via sponging miR-320a.

**Conclusion:**

Curcumin restrained proliferation and facilitated apoptosis in ovarian cancer by regulating the circ-PLEKHM3/miR-320a/SMG1 axis.

**Supplementary Information:**

The online version contains supplementary material available at 10.1186/s13048-021-00916-8.

## Introduction

Ovarian cancer is a global gynecological malignancy with ~ 90% cases as epithelial cancer [1], and is the fifth cause of cancer-associated death in women [2]. The standard treatments for this cancer include cytoreductive surgery and platinum-based chemotherapy [1]. Curcumin is the active component of turmeric which has an anti-cancerous property in multiple cancers, including ovarian cancer [3]. It can exhibit the anti-cancer role in ovarian cancer by decreasing tumorigenesis and increasing the efficiency of radio-chemotherapy [4, 5]. However, the mechanism underlying the anti-cancer role of curcumin in ovarian cancer remains largely unknown.

Non-coding RNA has been found to be the targets of curcumin in cancer therapy [6]. As a special kind of non-coding RNA, circular RNA (circRNAs) has attracted much attention in the role of cancer progression in recent years. Many studies have shown that circRNA may be a potential biomarker for the treatment and diagnosis of cancer [7, 8]. For example, circWHSC1 had been shown to promote ovarian cancer proliferation and metastasis [9], and circ_0078607 was found to be a tumour suppressor to inhibit ovarian cancer progression [10]. Circ_0001095 is located at chr2 and is derived from PLEKHM3 gene, also known as circ-PLEKHM3. In the past research, circ-PLEKHM3 was confirmed to be lowly expressed in ovarian cancer and have inhibitory effects on cell growth and metastasis [11]. Therefore, circ-PLEKHM3 might be a key target for the regulation of ovarian cancer progression.

A large number of studies have confirmed that circRNA can act as a microRNA (miRNA) sponge to indirectly regulate the expression of downstream genes [12]. MiRNAs have important roles in the development of ovarian cancer, and have been found to be regulated via curcumin [13, 14]. The previous report suggested that miR-320a was upregulated in paclitaxel-resistant ovarian cancer tissues and could accelerate ovarian cancer proliferation and invasion [15, 16]. Suppressor of morphogenesis in genitalia 1 (SMG1) is an important member of phosphoinositide 3-kinase related kinase family, which plays a tumor-suppressive role in human malignancies [17, 18]. More importantly, SMG1 had been found to repress ovarian cancer cell proliferation, migration and invasion [19].

In this study, we discovered that curcumin could regulate the expression of circ-PLEKHM3, miR-320a and SMG1. Additionally, the starBase (http://starbase.sysu.edu.cn/) software predicted that there had complementary binding sites between circ-PLEKHM3 and miR-320a, and miR-320a could combine with SMG1 3’UTR. However, it is not clear whether curcumin mediates ovarian cancer progression by regulating the circ-PLEKHM3/miR-320a/SMG1 axis. Our study wanted to study the mechanism of curcumin on cell proliferation and apoptosis in ovarian cancer through the circRNA network. This study might provide a new insight into understanding the activity of curcumin in ovarian cancer treatment.

## Materials and methods

### Patient tissues

The tumor tissues and paired normal samples were obtained via surgery from 35 ovarian cancer patients who did not receive other treatment in The Second Hospital of Dalian Medical University. All samples were maintained at − 80°C. The written informed consent was given via every subject. This work gained the approval of the ethics committee of The Second Hospital of Dalian Medical University.

### Cell culture and exposure to curcumin

Ovarian cancer cell lines [SKOV3 (Cat. No. CL-0215) and A2780 (Cat. No. CL-0013) cells] and human embryonic kidney 293 T cells (Cat. No. CL-0005) were provided by Procell Life Science Technology (Wuhan, China). Human ovarian surface epithelial cell line IOSE-80 cells (Cat. No. MZ-2207) were purchased from Mingzhou Biotechnology (Ningbo, China, https://www.mingzhoubio.com/goods-3560.html). All cells were grown in DMEM Medium (Gibco, Waltham, MA, USA) plus 10% FBS (Gibco) and 1% penicillin/streptomycin (Beyotime, Shanghai, china) under 5% CO_2_ at 37 °C. SKOV3 and A2780 cells were exposed to various doses (10, 20 or 40 μM) of curcumin (Sigma-Aldrich, St. Louis, MO, USA) for different times (24, 48 or 72 h). Cells treated by DMSO or PBS were used as control.

### Cell transfection

Circ-PLEKHM3 overexpression vector (oe-circ-PLEKHM3), SMG1 overexpression vector (oe-SMG1) and their control (vector) were constructed via Genomeditech (Shanghai, China). MiR-320a mimic and inhibitor or their controls (mimic NC and inhibitor NC), small interfering RNA for SMG1 (si-SMG1) and control (si-NC) were formed via Ribobio (Guangzhou, China). SKOV3 and A2780 cells were transfected with the constructed vectors (4.0 μg) or oligos (50 nM) with Lipofectamine 3000 (Invitrogen, Carlsbad, CA, USA).

### qRT-PCR

RNA was extracted by Trizol (Invitrogen), and then RNA was used for cDNA synthesis with the specific Reverse Transcriptase kit (Takara, Otsu, Japan). The cDNA (1:10 dilution) was used for qRT-PCR following mixing with SYBR (Invitrogen) and specific primers. The primer sequences were displayed in Table 1. U6 or GAPDH acted as normalized reference. Relative RNA level was calculated via 2^-ΔΔCt^ method.

**Table 1:** The primer sequences for qRT-PCR in this study

Name	Sequence (5′-3′)	
	Forward	Reverse
circ-PLEKHM3	GGAAGAACAAACGCCAATCT	TTTGGGAATGTCTGCTTGTG
miR-320a	GCCGAGAAAAGCTGGGTTGAGA	CTCAACTGGTGTCGTGGA
SMG1	GGAAGGTAATGAGCCGCAGA	TCAGTTCTGGGTTGCCAGTC
GAPDH	AGCCACATCGCTCAGACAC	GCCCAATACGACCAAATCC
U6	CTCGCTTCGGCAGCACATA	CGAATTTGCGTGTCATCCT

### Cell Counting Kit-8 (CCK-8)

1 × 10^4^ SKOV3 and A2780 cells were placed into 96-well plates. After treatment of different doses (10, 20 or 40 μM) of curcumin for 24, 48 or 72 h, each well was added with 10 μL CCK-8 reagent (Beyotime). Cells were cultured at 37°C for 3 h. The absorbance at 450 nm was detected with a microplate reader. Cell viability was calculated via normalizing to the control group at each time point × 100%.

### Colony formation analysis

Following transfection or treatment, SKOV3 and A2780 cells were inoculated in 6-well plates. After 12 days, cells were fixed via 4% paraformaldehyde (Beyotime), and stained with 0.5% crystal violet (Solarbio, Beijing, China). The visible colonies were imaged and counted.

### Flow cytometry

Cell apoptosis was measured with an Annexin V-FITC apoptosis detection kit (Sigma-Aldrich). 2 × 10^5^ SKOV3 and A2780 cells were inoculated in 12-well plates and cultured for 48 h. After that, cells were collected and staining with Annexin V-FITC and propidium iodide. The stained cells were detected with a flow cytometer. The apoptotic rate represented a percentage of apoptotic cells (Annexin V^+^/PI^−^ and Annexin V^+^/PI^+^).

### Western blot

Protein was isolated with a total protein extraction kit (Applygen, Beijing, China), and quantified with a BCA kit (Solarbio). Thity microgram protein was separated via SDS-PAGE gel and transferred on PVDF membrane (Beyotime). The membrane was blocked with non-fat milk and incubated with antibodies for cleaved caspase-3 (c-caspase-3) (ab32042, 1:500, Abcam, Cambridge, CA, USA), PCNA (ab92552, 1:5000, Abcam), Bax (ab262929, 1:2000, Abcam), SMG1 (ab151730, 1:300, Abcam) or GAPDH (ab181602, 1:3000, Abcam) overnight and HRP-labeled IgG (ab205722, 1:10000, Abcam) for 2 h. GAPDH acted as a loading control. Following interacting with enhanced chemiluminescence reagent (Solarbio), the blots were analyzed via Quantity One software.

### Murine xenograft model

Total of 20 BALB/c athymic mice (female, 5-week-old, ~ 20 g) were purchased from Charles River Laboratories (Beijing, China), and housed in specific pathogen-free microisolator cages. A2780 cells (3 × 10^6^) transfected with oe-circ-PLEKHM3 or lentiviral vector were injected into mice (*n* = 5/group) via subcutaneous inoculation. After 1 week, mice were intraperitoneally injected with 15 mg/kg of curcumin or equal volume of DMSO every 2 days. Tumor volume was monitored every week, and analyzed according to the formula volume = 0.5 × length × width^2^. After 5 weeks, mice were euthanized using 5% isoflurane. Tumor tissues were collected and weighed. The animal experiments were in line with the guidelines of the National Institutes of Health guide for the Care and Use of Laboratory animals (NIH Publications No. 8023, revised 1978), and approved via the Institutional Animal Care and Use Committee of The Second Hospital of Dalian Medical University.

### Dual-luciferase reporter assay and RIP assay

The target site of miR-320a and circ-PLEKHM3 or SMG1 3’UTR was searched via starBase. The sequence of circ-PLEKHM3 or SMG1 3’UTR containing miR-320a complementary site was cloned in pGL3 vector (Promega, Madison, WI, USA), generating the luciferase report vector circ-PLEKHM3 wt or SMG1 3’UTR wt. The mutant-type sequence containing mutant site was used to construct luciferase reporter vector circ-PLEKHM3 mut or SMG1 3’UTR mut based on the pGL3 vector. These luciferase reporter vectors and miR-320a mimic or mimic NC were co-transfected into 293 T cells. After 24 h, the luciferase activity was examined with luciferase analysis kit (Promega).

RIP analysis was carried out using a Magna RIP kit (Sigma-Aldrich). 1 × 10^7^ SKOV3 and A2780 cells were lysed, and the lysates were interacted with the beads pre-coated with anti-Ago2 or anti-IgG overnight at 4°C. RNA enriched on beads was isolated, and circ-PLEKHM3 and miR-320a enrichment levels were analyzed via qRT-PCR.

### Statistical analysis

The experiments were conducted 3 times. Data were shown as mean ± SD, and tested via GraphPad Prism 8 (GraphPad Inc., La Jolla, CA, USA). The linear correlation was analyzed by Pearson test. The comparison of 2 groups was determined by Student *t*-test. The analysis of variance with Tukey’s post hoc test was exploited for the comparison of multiple groups. It was significant when *P* < 0.05.

## Results

### Curcumin constrained proliferation and promoted apoptosis in ovarian cancer cells

**Fig. 1:**
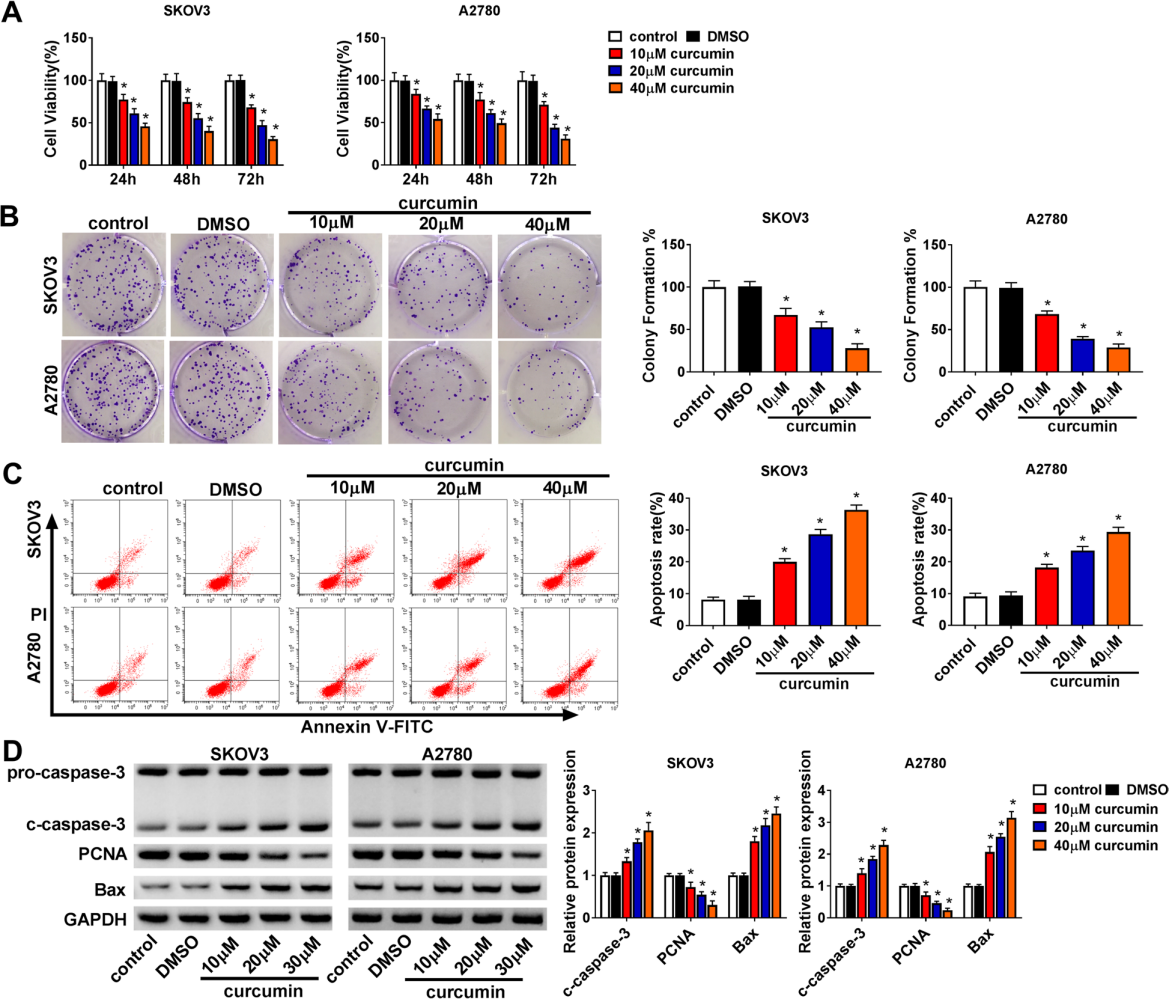
The effect of curcumin on cell proliferation and apoptosis in ovarian cancer cells. Cell viability (**A**), colony-formation ability (**B**), apoptosis (**C**), and protein levels of c-caspase-3, PCNA and Bax (**D**) were detected in SKOV3 and A2780 cells after exposure to various doses of curcumin. ^*^P < 0.05

To study the function of curcumin on ovarian cancer progression, SKOV3 and A2780 cells were stimulated via various doses of curcumin. Exposure to curcumin significantly decreased the viability of SKOV3 and A2780 cells at 24, 48 and 72 h in a dose-dependent manner when comparing to DMSO or control group (Fig. 1A). Moreover, curcumin evidently inhibited the colony-formation ability of SKOV3 and A2780 cells in comparison to DMSO or control group in a dose-dependent pattern (Fig. 1B). Additionally, curcumin clearly caused the apoptosis of SKOV3 and A2780 cells compared with DMSO or control group in a dose-dependent pattern (Fig. 1C). Furthermore, the pro-apoptotic protein (c-caspase-3 and Bax) and proliferation protein (PCNA) levels were detected. Results showed that curcumin led to obvious upregulation of c-caspase-3 and Bax, and reduction of PCNA in SKOV3 and A2780 cells (Fig. 1D). These data indicated that curcumin suppressed ovarian cancer progression in vitro.

### Circ-PLEKHM3 aggravated the effect of curcumin on proliferation and apoptosis in ovarian cancer cells

**Fig. 2:**
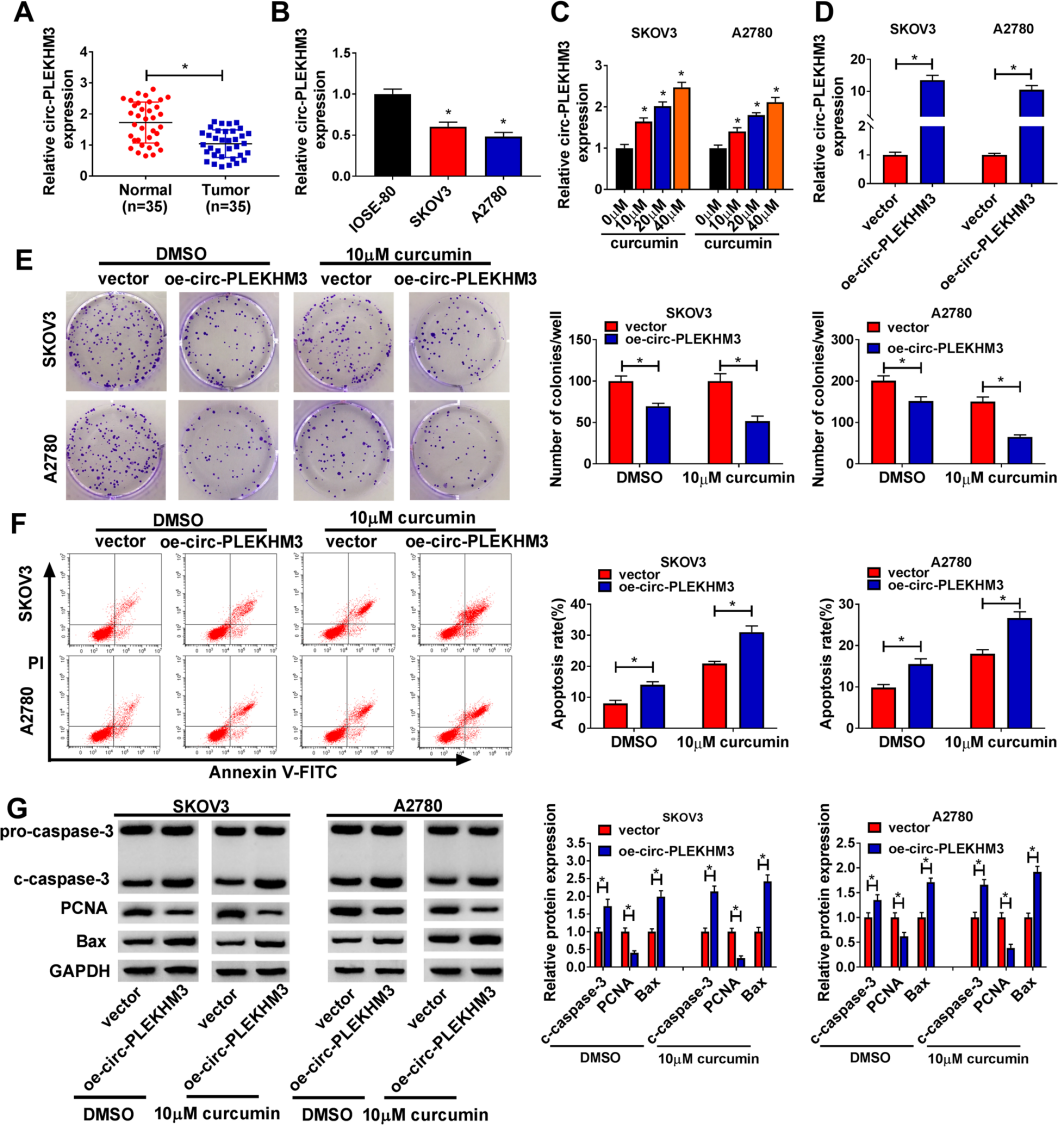
The influence of circ-PLEKHM3 on the proliferation and apoptosis in curcumin-treated ovarian cancer cells. **A** Circ-PLEKHM3 expression was examined in tumor and normal tissues (*n* = 35). **B** Circ-PLEKHM3 level was measured in SKOV3, A2780 and IOSE-80 cells. **C** Circ-PLEKHM3 abundance was examined in SKOV3 and A2780 cells after stimulation of various concentrations of curcumin. **D** Circ-PLEKHM3 level was detected in cells with transfection of oe-circ-PLEKHM3 or vector. Colony-formation ability (**E**), apoptosis (**F**), and protein levels of c-caspase-3, PCNA and Bax (**G**) were examined in cells with transfection of oe-circ-PLEKHM3 or vector under the treatment of curcumin or DMSO. ^*^P < 0.05

To analyze whether circ-PLEKHM3 was needed for curcumin in regulating ovarian cancer progression, circ-PLEKHM3 expression was firstly measured in ovarian cancer. Low level of circ-PLEKHM3 was measured in ovarian cancer samples in comparison to normal tissues (Fig. 2A). Moreover, circ-PLEKHM3 level was significantly reduced in SKOV3 and A2780 cells in comparison to IOSE-80 cells (Fig. 2B). Exposure to curcumin markedly upregulated circ-PLEKHM3 abundance in SKOV3 and A2780 cells in a dose-dependent pattern (Fig. 2C). After transfected with oe-circ-PLEKHM3 into SKOV3 and A2780 cells, we confirmed that circ-PLEKHM3 expression was indeed increased (Fig. 2D). Function analysis showed that circ-PLEKHM3 overexpression significantly reduced the colony-formation ability of SKOV3 and A2780 cells under treatment of DMSO or curcumin (Fig. 2E). Additionally, circ-PLEKHM3 upregulation evidently promoted cell apoptosis under treatment of DMSO or curcumin (Fig. 2F). Besides, circ-PLEKHM3 overexpression obviously enhanced the c-caspase-3 and Bax protein levels, and decreased the PCNA protein level in SKOV3 and A2780 cells under the presence of DMSO or curcumin (Fig. 2G). These data suggested that curcumin inhibited ovarian cancer progression via increasing circ-PLEKHM3 in vitro.

### Circ-PLEKHM3 overexpression exacerbated curcumin-mediated tumor growth suppression in ovarian cancer

**Fig. 3:**
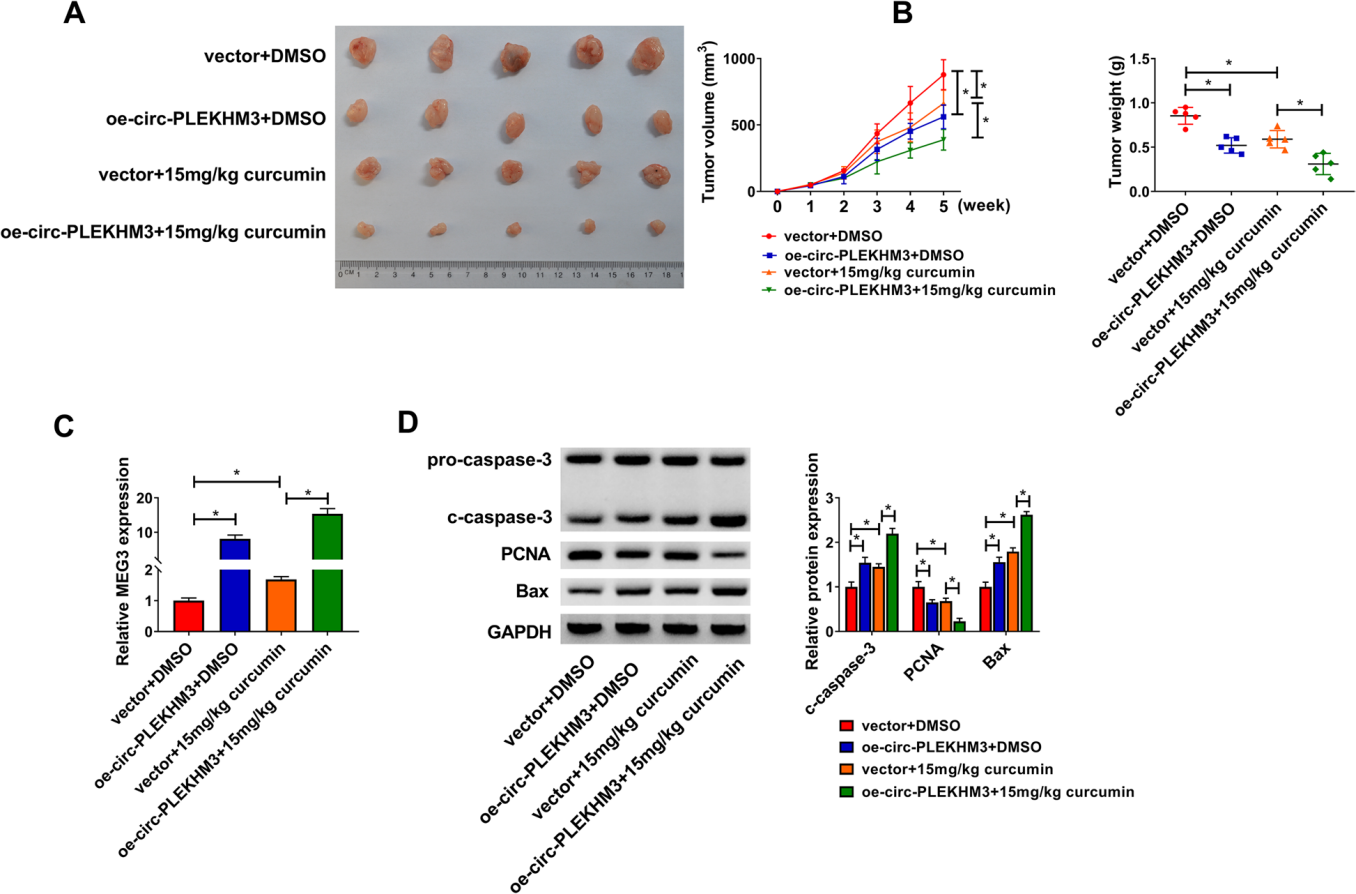
The influence of circ-PLEKHM3 and curcumin on tumor growth in ovarian cancer. A2780 cells transfected with oe-circ-PLEKHM3 or vector were injected into nude mice, and next mice were subjected to curcumin or DMSO. Tumor volume (**A**), weight (**B**), circ-PLEKHM3 expression (**C**), and protein abundances of c-caspase-3, PCNA and Bax (**D**) were detected in each group. ^*^P < 0.05

To assess whether circ-PLEKHM3 could affect the function of curcumin in ovarian cancer in vivo, A2780 cells with stably expressing circ-PLEKHM3 were used for the establishment of xenograft model, and next the murine model was treated with curcumin or DMSO. As exhibited in Fig. 3A and B, circ-PLEKHM3 overexpression clearly reduced tumor volume and weight, and it deteriorated curcumin-mediated loss of tumor growth. Moreover, higher level of circ-PLEKHM3 was detected in xenograft tumor tissues in the transfection of oe-circ-PLEKHM3 group in the presence of DMSO or curcumin (Fig. 3C). In addition, circ-PLEKHM3 overexpression enhanced curcumin-mediated promotion of c-caspase-3 and Bax protein expression, and reduction of PCNA protein expression in tumor tissues (Fig. 3D). These results showed that curcumin repressed ovarian cancer growth by regulating circ-PLEKHM3 in vivo.

### MiR-320a was targeted by circ-PLEKHM3

**Fig. 4:**
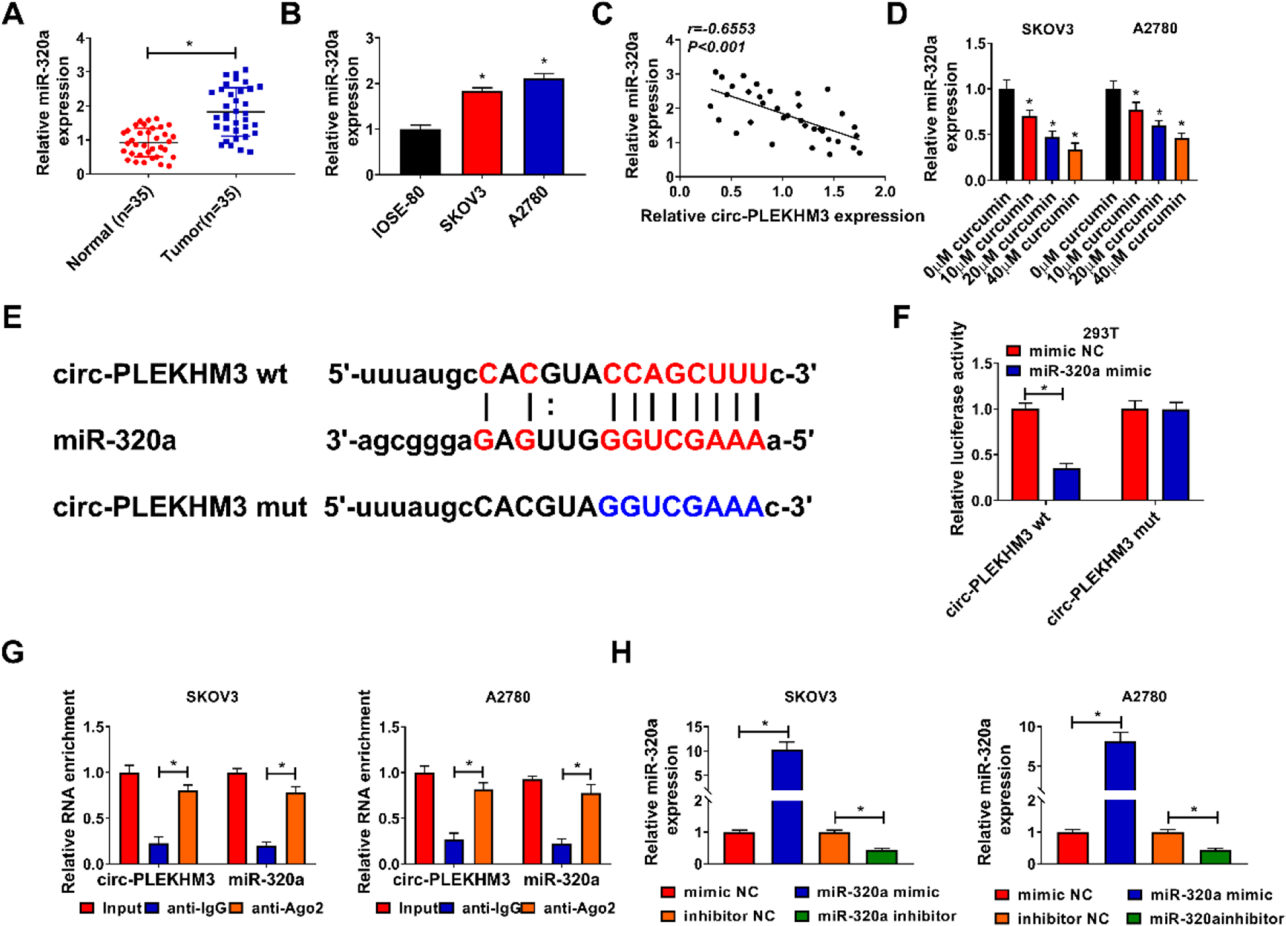
Circ-PLEKHM3 sponged miR-320a. **A** MiR-320a expression was detected in tumor and normal tissues (*n* = 35). **B** MiR-320a level was measured in SKOV3, A2780 and IOSE-80 cells. **C** The linear correlation of miR-320a and circ-PLEKHM3 in ovarian cancer tissues was analyzed. **D** MiR-320a abundance was examined in SKOV3 and A2780 cells after exposure to various concentrations of curcumin. **E** The binding site of circ-PLEKHM3 and miR-320a was predicted via starBase. **F** Luciferase activity was detected in 293 T cells with transfection of circ-PLEKHM3 wt or mut and miR-320a mimic or mimic NC. **G** Circ-PLEKHM3 and miR-320a abundances were detected after RIP. **H** MiR-320a abundance was examined in SKOV3 and A2780 cells transfected with mimic NC, miR-320a mimic, inhibitor NC or miR-320a inhibitor. ^*^P < 0.05

MiR-320a was found to be evidently upregulated in ovarian cancer tissues and cells (Fig. 4A and B), and its expression level in ovarian cancer tissues was negatively correlated with circ-PLEKHM3 expression (Fig. 4C). Moreover, miR-320a abundance was obviously reduced via curcumin in a dose-dependent pattern (Fig. 4D). Using the starBase software, we found that there had binding sites between miR-320a and circ-PLEKHM3 (Fig. 4E). To confirm the association of miR-320a and circ-PLEKHM3, dual-luciferase reporter analysis was conducted in 293 T cells. MiR-320a overexpression induced 65% reduction of luciferase activity in the circ-PLEKHM3 wt group, but it showed little influence in the circ-PLEKHM3 mut group (Fig. 4F). In addition, RIP analysis showed circ-PLEKHM3 and miR-320a were enriched in anti-Ago2 (Fig. 4G). These data indicated that miR-320a could be sponged by circ-PLEKHM3 in ovarian cancer cells. Moreover, the efficacy of miR-320a mimic or inhibitor was validated in Fig. 4H.

### MiR-320a overexpression reversed the regulation of circ-PLEKHM3 on curcumin-induced ovarian cancer cells progression

**Fig. 5:**
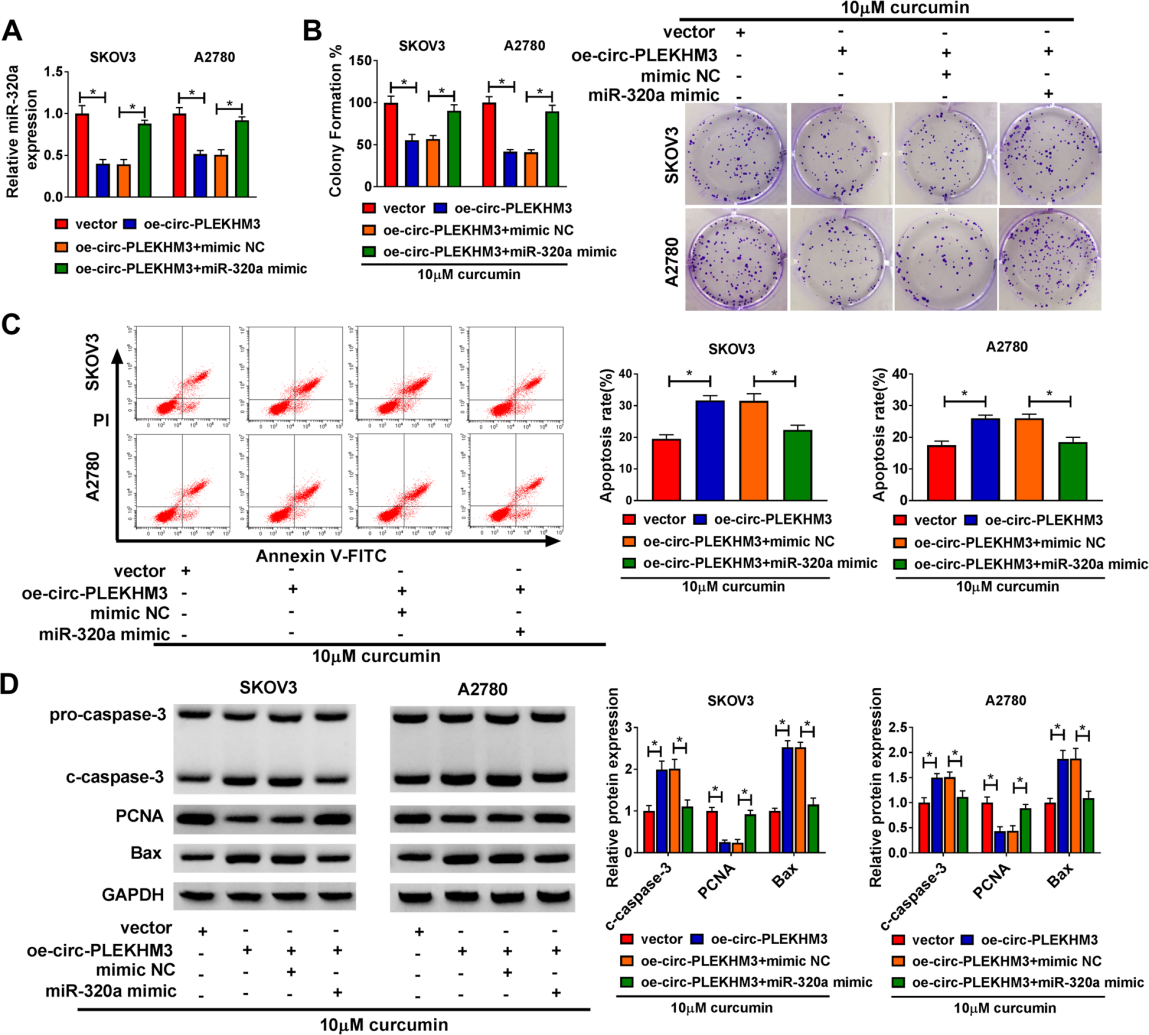
The effect of miR-320a on circ-PLEKHM3-mediated ovarian cancer progression under curcumin exposure. **A** MiR-320a abundance was measured in cells transfected with vector, oe-circ-PLEKHM3, oe-circ-PLEKHM3 + mimic NC or miR-320a mimic. Colony-formation ability (**B**), apoptosis (**C**), and protein levels of c-caspase-3, PCNA and Bax (**D**) were determined in cells with transfection of vector, oe-circ-PLEKHM3, oe-circ-PLEKHM3 + mimic NC or miR-320a mimic after exposure to curcumin. ^*^P < 0.05

To explore whether miR-320a was required for circ-PLEKHM3 during the process of curcumin regulated ovarian cancer progression, SKOV3 and A2780 cells were transfected with oe-circ-PLEKHM3 and miR-320a mimic. MiR-320a abundance was evidently reduced by circ-PLEKHM3 overexpression in SKOV3 and A2780 cells, which was restored via introduction of miR-320a mimic (Fig. 5A). After treated with curcumin, we found that miR-320a addition mitigated circ-PLEKHM3-mediated suppression of colony-formation ability in SKOV3 and A2780 cells (Fig. 5B). Additionally, miR-320a upregulation weakened the promotion of circ-PLEKHM3 on the apoptosis of SKOV3 and A2780 cells in the presence of curcumin (Fig. 5C). Furthermore, miR-320a overexpression reversed the increasing effect of circ-PLEKHM3 on the c-caspase-3 and Bax protein expression, and the decreasing effect on PCNA protein expression in SKOV3 and A2780 cells under the treatment with curcumin (Fig. 5D). These results suggested that circ-PLEKHM3 mediated the anti-cancer role of curcumin in ovarian cancer via modulating miR-320a.

### SMG1 was a target of miR-320a

**Fig. 6:**
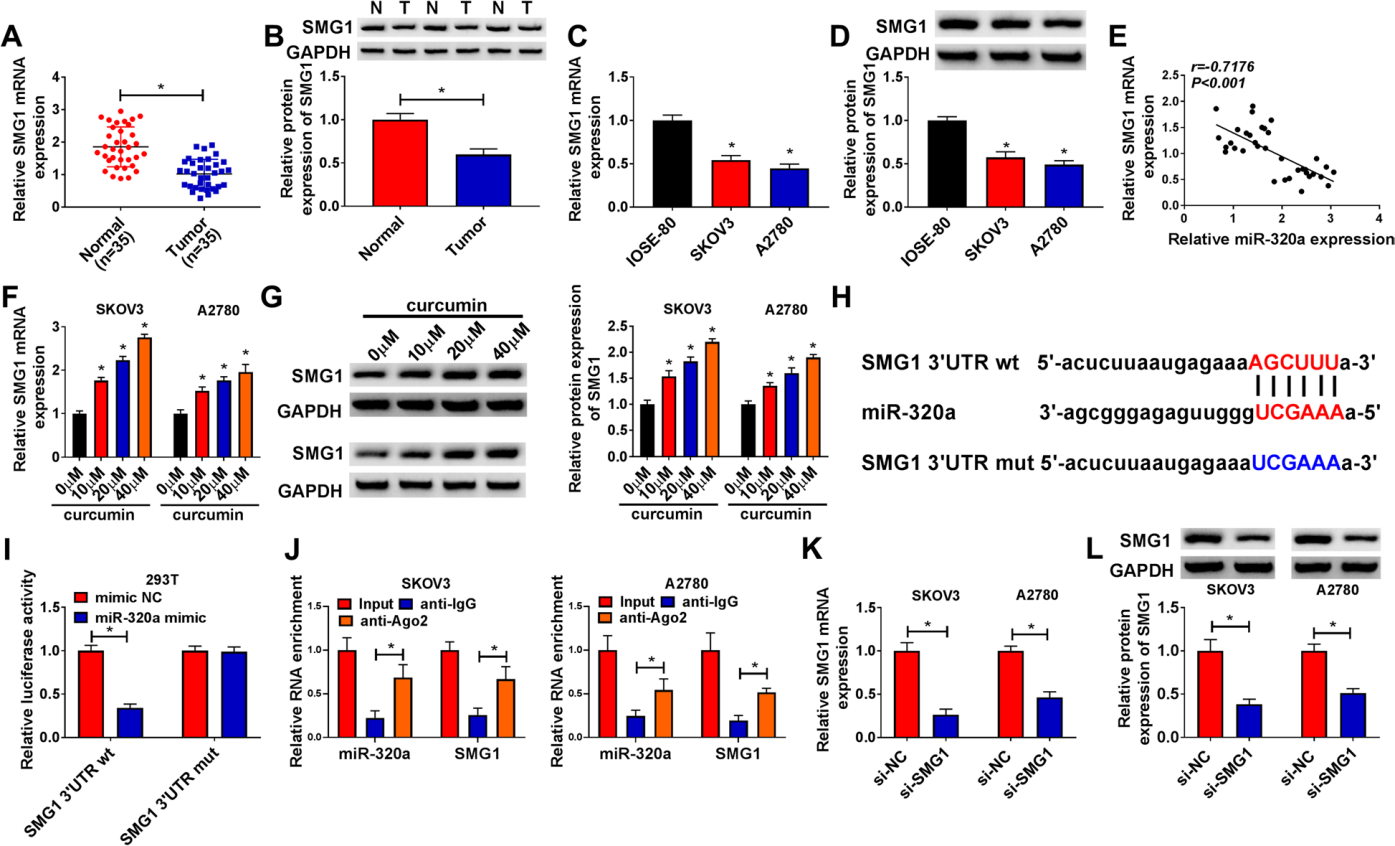
SMG1 was a target of miR-320a. **A and B** SMG1 level was measured in tumor and normal tissues (*n* = 35). **C and D** SMG1 level was examined in SKOV3, A2780 and IOSE-80 cells. **E** The linear correlation of miR-320a and SMG1 in ovarian cancer tissues was analyzed. **F and G** SMG1 abundance was detected in SKOV3 and A2780 cells after exposure to various concentrations of curcumin. **H** The binding site of miR-320a and SMG1 was predicted via starBase. **I** Luciferase activity was detected in 293 T cells transfected with SMG1 3’UTR wt or mut and miR-320a mimic or mimic NC. **J** The interaction between miR-320a and SMG1 was analyzed by RIP assay. **K-L** SMG1 abundance was measured in SKOV3 and A2780 cells transfected with si-NC or si-SMG1. ^*^P < 0.05

SMG1 expression was discovered to be significantly reduced in ovarian cancer tissues and cells (Fig. 6A-D), and its expression in ovarian cancer tissues was negatively associated with miR-320a level (Fig. 6E). Under the treatment with curcumin, SMG1 expression also was evidently elevated in SKOV3 and A2780 cells in a dose-dependent pattern (Fig. 6F and G). To our surprise, starBase software analysis showed that miR-320a could combine with SMG1 3’UTR in a complementary way (Fig. 6H). To validate the relationship of miR-320a and SMG1, the dual-luciferase reporter analysis was performed in 293 T cells. MiR-320a addition led to 66% loss of luciferase activity in the SMG1 3’UTR wt group, while it did not alter the activity in the SMG1 3’UTR mut group (Fig. 6I). RIP analysis showed SMG1 and miR-320a were markedly enriched in anti-Ago2 RIP group (Fig. 6J). These results indicated that SMG1 could be targeted by miR-320a in ovarian cancer cells. Furthermore, we constructed the siRNA of SMG1 and confirmed that si-SMG1 indeed decreased SMG1 expression in SKOV3 and A2780 cells at the mRNA level and protein level (Fig. 6K and L). Additionally, oe-SMG1 transfection elevated SMG1 mRNA and protein levels in SKOV3 and A2780 cells compared to vector control groups (Fig. S1A and B). As suggested by colony formation assay and flow cytometry analysis, SMG1 overexpression repressed cell colony formation and promoted cell apoptosis in curcumin-treated SKOV3 and A2780 cells (Fig. S1C and D).

### MiR-320a knockdown inhibited curcumin-treated ovarian cancer progression by increasing SMG1

**Fig. 7:**
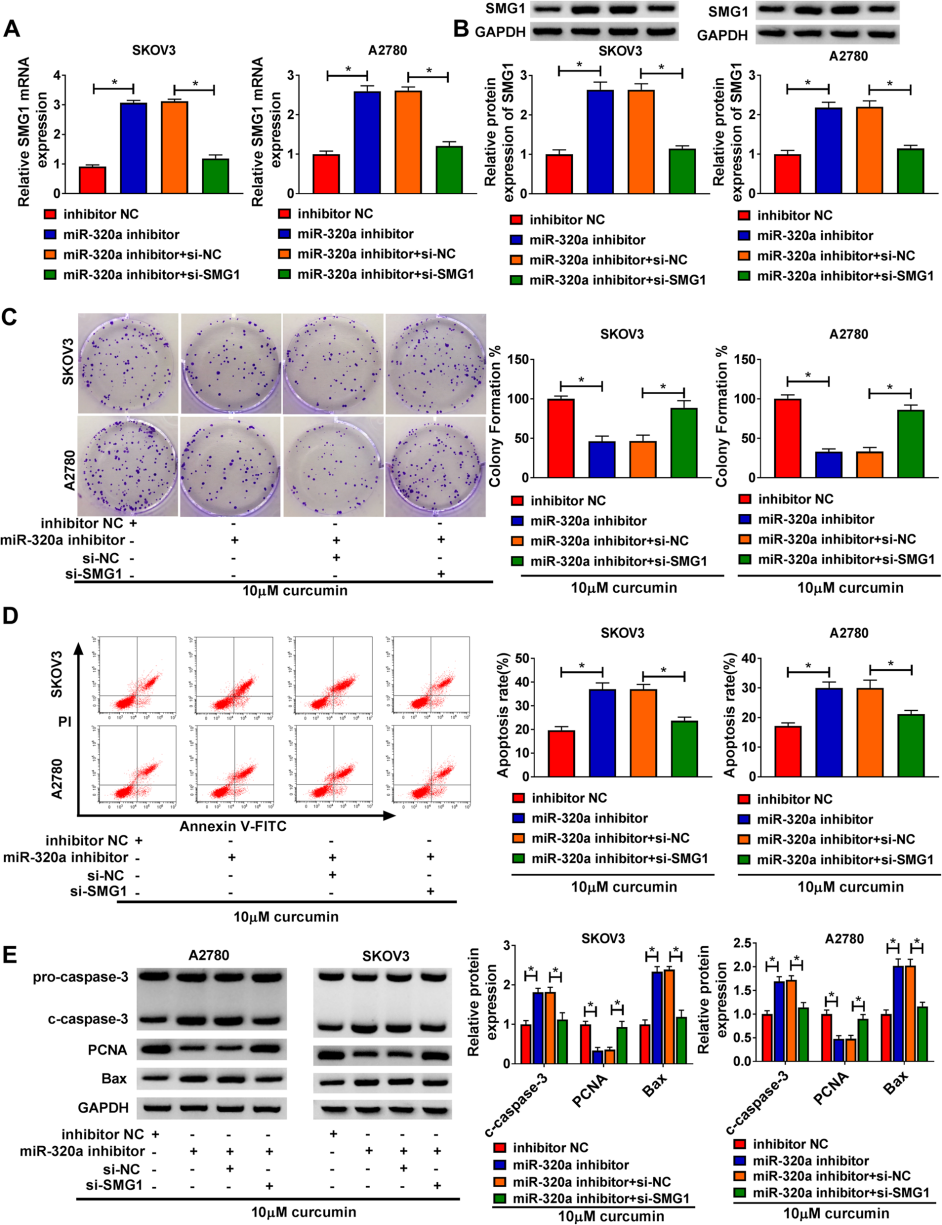
The influence of miR-320a and SMG1 on ovarian cancer progression under curcumin exposure. **A and B** SMG1 expression was detected in SKOV3 and A2780 cells with transfection of inhibitor NC, miR-320a inhibitor, miR-320a inhibitor + si-NC or si-SMG1. Colony-formation ability (**C)**, apoptosis (**D**), and protein levels of c-caspase-3, PCNA and Bax (**E**) were measured in SKOV3 and A2780 cells with transfection of inhibitor NC, miR-320a inhibitor, miR-320a inhibitor + si-NC or si-SMG1 after exposure to curcumin. ^*^P < 0.05

To study the function of miR-320a/SMG1 axis on regulating the role of curcumin in ovarian cancer progression, SKOV3 and A2780 cells were transfected with miR-320a inhibitor and si-SMG1. By detecting SMG1 expression, we found that SMG1 mRNA and protein expression was significantly enhanced by miR-320a knockdown in SKOV3 and A2780 cells, which was weakened via the addition of si-SMG1 (Fig. 7A and B). Moreover, miR-320a down-regulation evidently restrained the colony-formation ability in curcumin-treated cells, which was abolished via SMG1 silencing (Fig. 7C). In addition, miR-320a knockdown clearly promoted the apoptosis of SKOV3 and A2780 cells in the presence of curcumin, and this effect could be reversed by SMG1 interference (Fig. 7D). Furthermore, miR-320a knockdown could promote the protein levels of c-caspase-3 and Bax, and reduce the protein level of PCNA in curcumin-treated SKOV3 and A2780 cells, which these effects also could be abolished by SMG1 inhibition (Fig. 7E). These data showed that miR-320a knockdown enhanced the anti-cancer role of curcumin in ovarian cancer via regulating SMG1.

### Circ-PLEKHM3 positively regulated SMG1 expression via sponging miR-320a

**Fig. 8:**
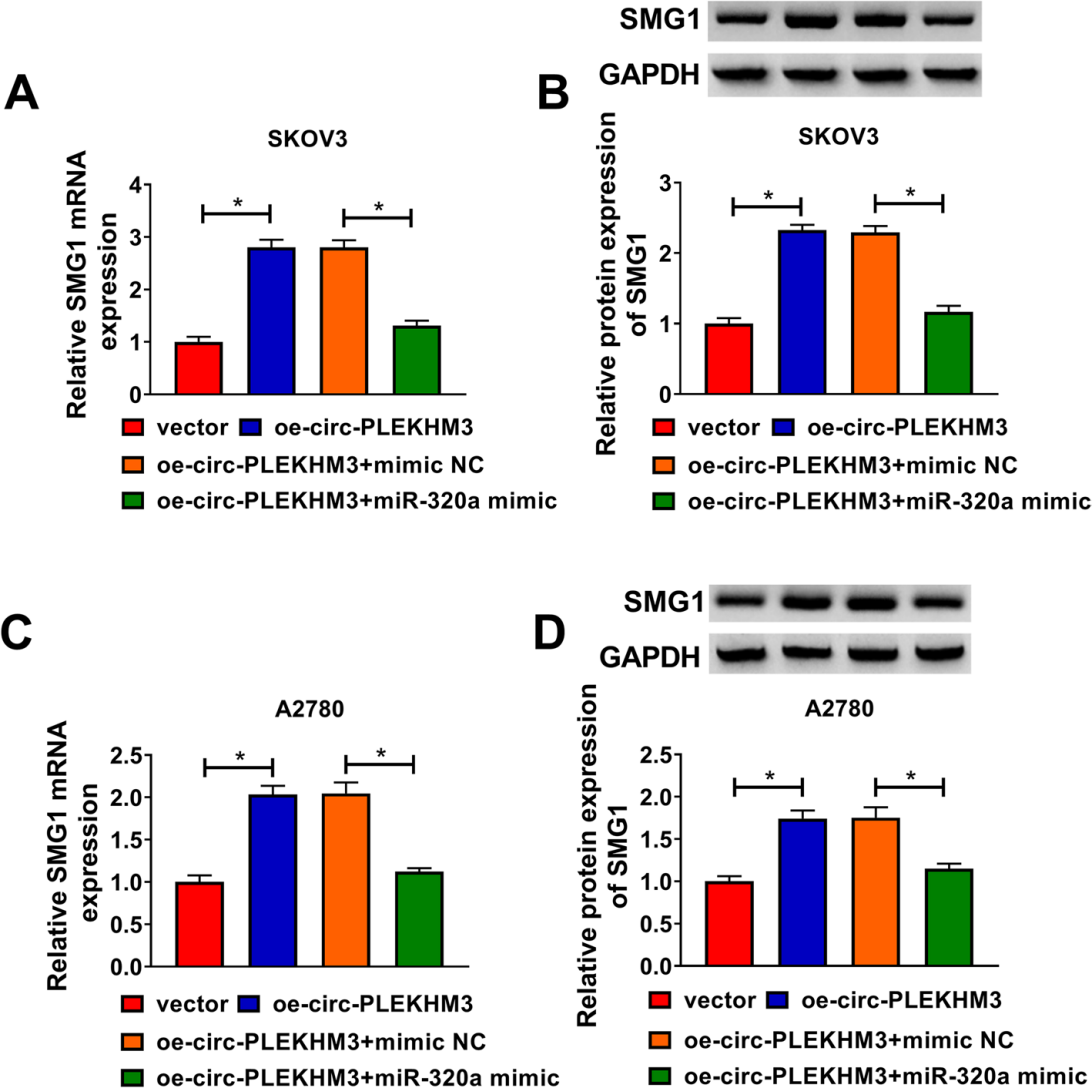
The influence of circ-PLEKHM3/miR-320a axis on SMG1 expression in ovarian cancer cells. **A-D** SMG1 abundance was detected in cells transfected with vector, oe-circ-PLEKHM3, oe-circ-PLEKHM3 + mimic NC or miR-320a mimic. ^*^P < 0.05

To explore whether SMG1 could be regulated via circ-PLEKHM3/miR-320a axis in ovarian cancer cells, we measured SMG1 expression in SKOV3 and A2780 cells co-transfected with oe-circ-PLEKHM3 and miR-320a mimic. As displayed in Fig. 8A-D, SMG1 mRNA and protein levels were significantly increased by circ-PLEKHM3 overexpression, which this effect could be weakened by miR-320a overexpression. These data revealed that circ-PLEKHM3 upregulated SMG1 by sponging miR-320a in ovarian cancer cells.

## Discussion

Ovarian cancer is a common gynecological tumor that is usually diagnosed at an advanced stage and has a low 5-year overall survival [20]. Curcumin exhibits the important anti-cancer activity in ovarian cancer via the pro-apoptotic function [5, 21]. In this study, we studied the function and potential mechanism of curcumin in ovarian cancer development. Here, we found that curcumin inhibited ovarian cancer cell proliferation and promoted apoptosis, and first confirmed it was associated with the regulatory network of circ-PLEKHM3/miR-320a/SMG1.

Liu et al. reported that curcumin could constrain ovarian cancer cell proliferation and facilitate apoptosis by inhibiting autophagy and AKT/mTOR/p70S6 pathway [22]. Yen et al. suggested that curcumin could suppress ovarian cancer cell colony formation via blocking the Wnt/β-catenin pathway [23]. These reports indicated the anti-cancer property of curcumin in ovarian cancer treatment. Similarly, we also confirmed the anti-cancer function of curcumin in ovarian cancer. In the past research, curcumin had been found to improve the radiosensitization of nasopharyngeal carcinoma through regulating the circRNA network [24, 25]. Xu et al. suggested that curcumin could suppress non-small cell lung cancer progression by regulating circ-PRKCA [26]. However, it is not clear whether curcumin also mediated ovarian cancer progression by regulating circRNA networks. Here, we found that circ-PLEKHM3 was downregulated in ovarian cancer, and its expression could be promoted by curcumin. Function analysis showed that circ-PLEKHM3 overexpression could aggravate curcumin function by suppressing cell proliferation, triggering apoptosis and reducing tumorigenesis in ovarian cancer. These data revealed that curcumin might regulate ovarian cancer progression by promoting circ-PLEKHM3. In addition, the anti-cancer role of circ-PLEKHM3 was confirmed in our study, which was consistent with the previous study [11].

A previous report displayed the circ-PLEKHM3 acted as miR-9 sponge to regulate ovarian cancer progression [11]. The regulatory network was complex, and we wanted to explore an additional network mediated via circ-PLEKHM3 in ovarian cancer. In this, miR-320a was found to be targeted by circ-PLEKHM3. Multiple reports indicated that miR-320a usually functioned as an oncogenic miRNA in human tumor, such as retinoblastoma [27], prostate cancer [28] and colorectal cancer [29]. Here, miR-320a mimic reversed the regulation of circ-PLEKHM3 on curcumin-mediated ovarian cancer cell proliferation and apoptosis, further confirming that circ-PLEKHM3 sponged miR-320a to participate in ovarian cancer progression. Our study also validated the carcinogenic role of miR-320a in ovarian cancer, which was consistent with previous reports [15, 16]. These data indicated the importance of circ-PLEKHM3/miR-320a axis for curcumin in ovarian cancer development.

Next, we further analyzed the downstream target of miR-320a, and confirmed SMG1 was targeted via miR-320a. SMG1 was reported to play a tumor-suppressive function in tumors, like acute myeloid leukemia, gastric carcinogenesis, hepatocellular carcinoma and nasopharyngeal carcinoma [17, 18, 30, 31]. Furthermore, Zeng et al. reported SMG1 could repress ovarian cancer cell proliferation and motility [19]. These reports indicated the anti-cancer role of SMG1 in ovarian cancer. Here, we also confirmed that SMG1 expression was positively regulated by circ-PLEKHM3 and negatively regulated by miR-320a. In addition, we found that curcumin could up-regulate SMG1 expression via modulating circ-PLEKHM3/miR-320a axis.

In conclusion, curcumin could suppress proliferation and promote apoptosis in ovarian cancer, possibly via regulating circ-PLEKHM3/miR-320a/SMG1 axis. This research might propose a novel mechanism for understanding the function of curcumin in ovarian cancer.

## Supplementary Information

**Additional file 1: Figure S1.** SMG1 overexpression suppressed cell viability and promoted cell viability in curcumin-treated ovarian cancer cells. (A and B) The mRNA and protein levels of SMG1 in SKOV3 and A2780 cells transfected with vector or oe-SMG1 were measured by qRT-PCR and western blot assay. (C and D) After oe-SMG1 or vector transfection and curcumin treatment, cell colony formation and cell apoptosis were analyzed. ^*^P < 0.05.

**Additional file 2.**

**Additional file 3.**

**Additional file 4.**

## Abbreviations

circRNA: Circular RNA
microRNA: miRNA
SMG1: Suppressor of morphogenesis in genitalia 1
NC: Negative control
CCK-8: Cell counting kit-8
