# Supplementary figures and images for: Curcumin inhibits ovarian cancer progression by regulating circ-PLEKHM3/miR-320a/SMG1 axis
Source: J Ovarian Res. 2021 Nov 16;14:158. doi: 10.1186/s13048-021-00916-8 (PMC8594156; doi:10.1186/s13048-021-00916-8)

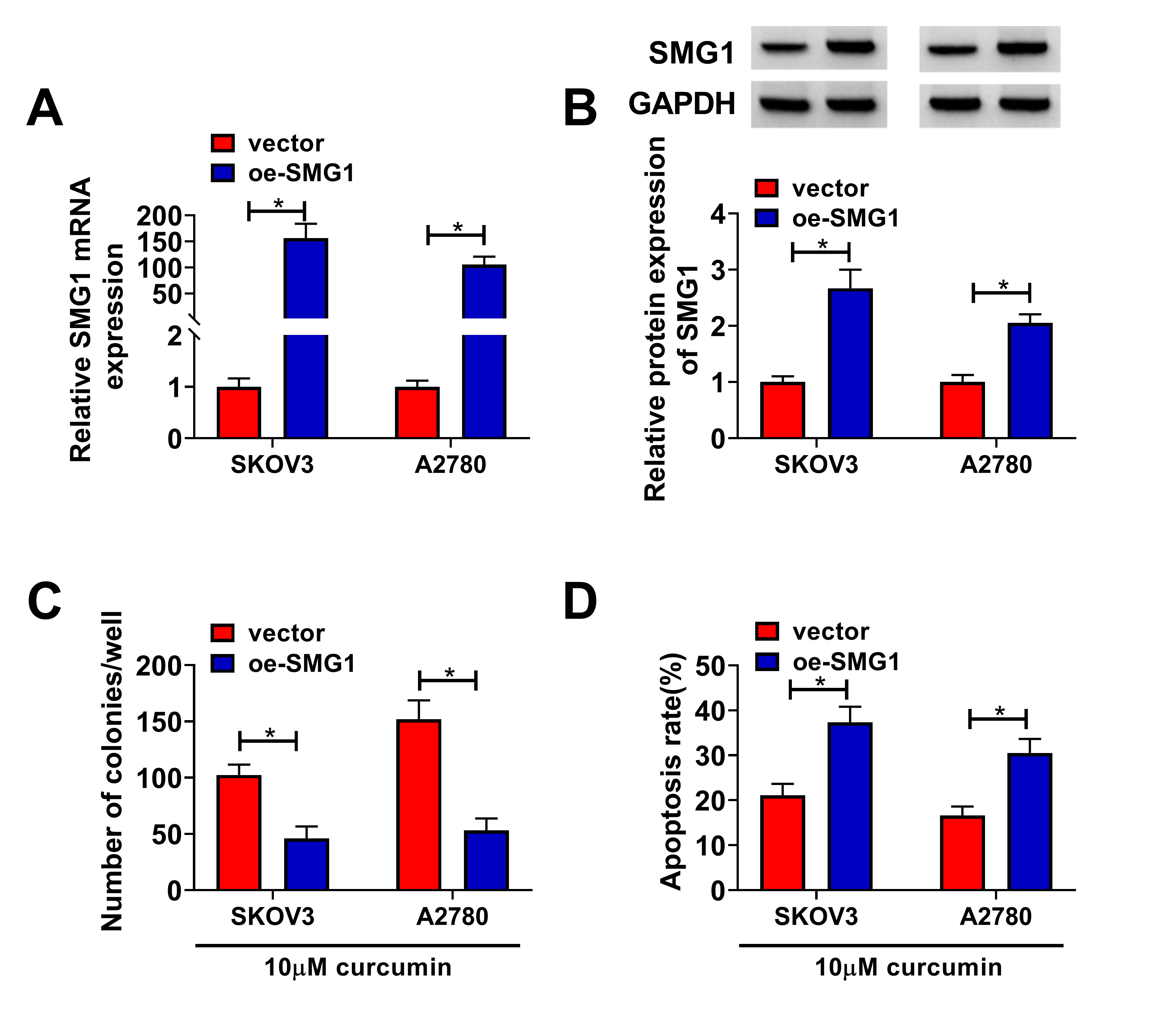

Supplement: Supplementary file 1 — Additional file 1: Figure S1. SMG1 overexpression suppressed cell viability and promoted cell viability in curcumin-treated ovarian cancer cells. (A and B) The mRNA and protein levels of SMG1 in SKOV3 and A2780 cells transfected with vector or oe-SMG1 were measured by qRT-PCR and western blot assay. (C and D) After oe-SMG1 or vector transfection and curcumin treatment, cell colony formation and cell apoptosis were analyzed. *P < 0.05. [file 13048_2021_916_MOESM1_ESM.tif]

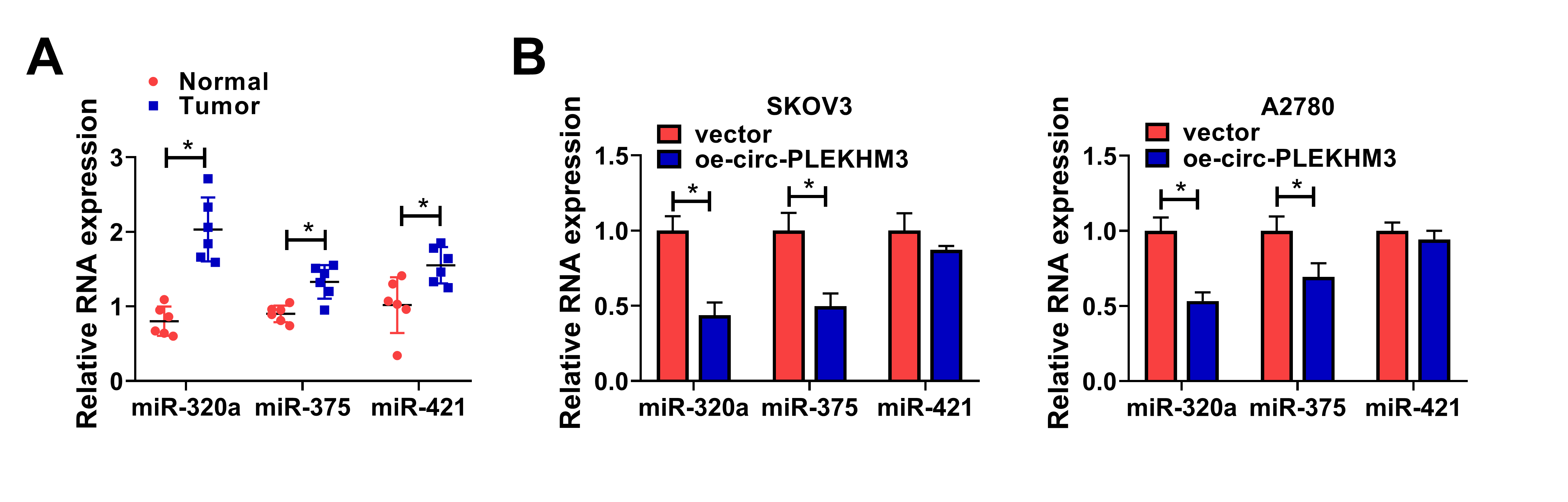

Supplement: Supplementary file 2 — Additional file 2. [file 13048_2021_916_MOESM2_ESM.tif]

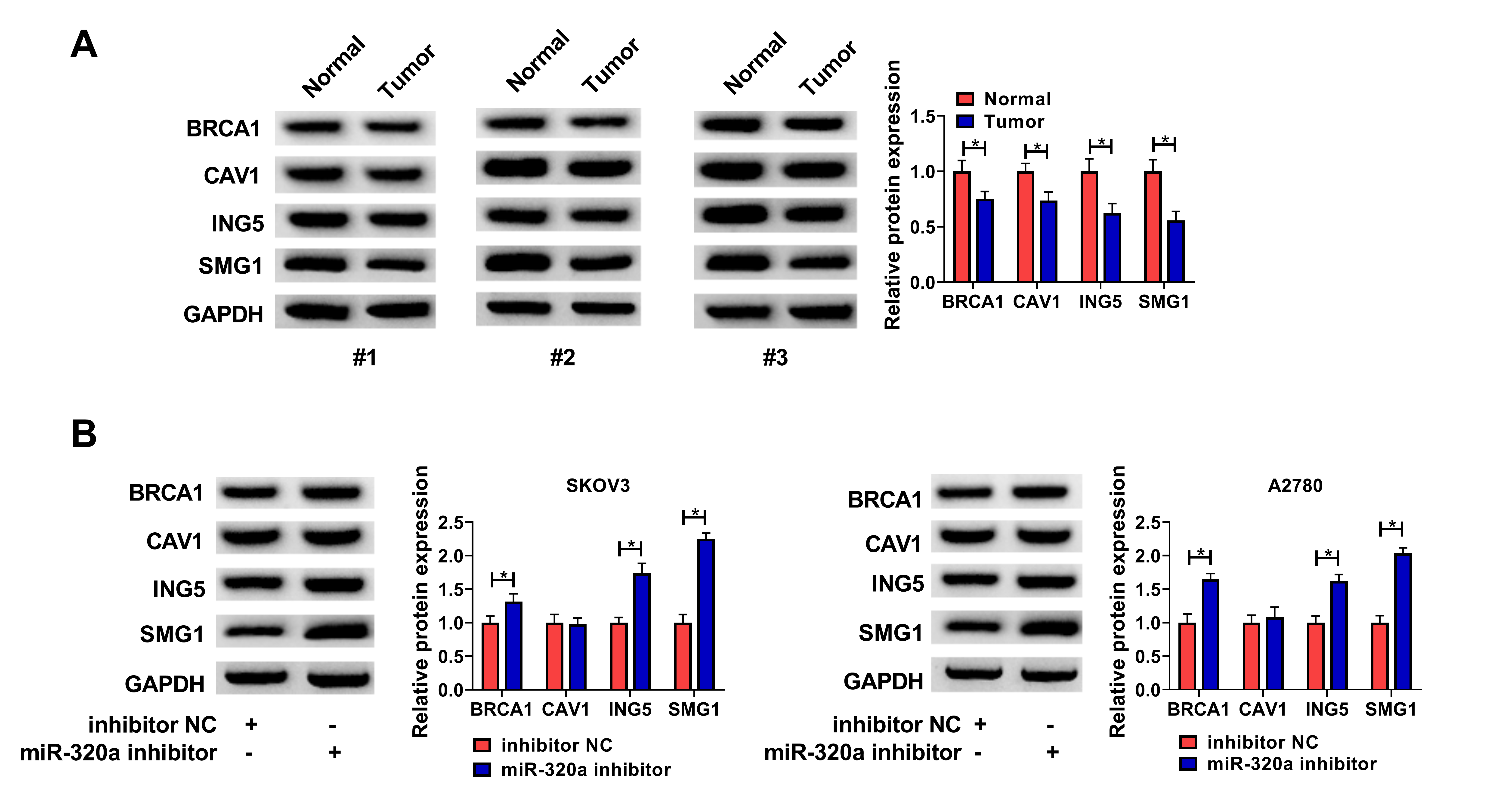

Supplement: Supplementary file 3 — Additional file 3. [file 13048_2021_916_MOESM3_ESM.tif]

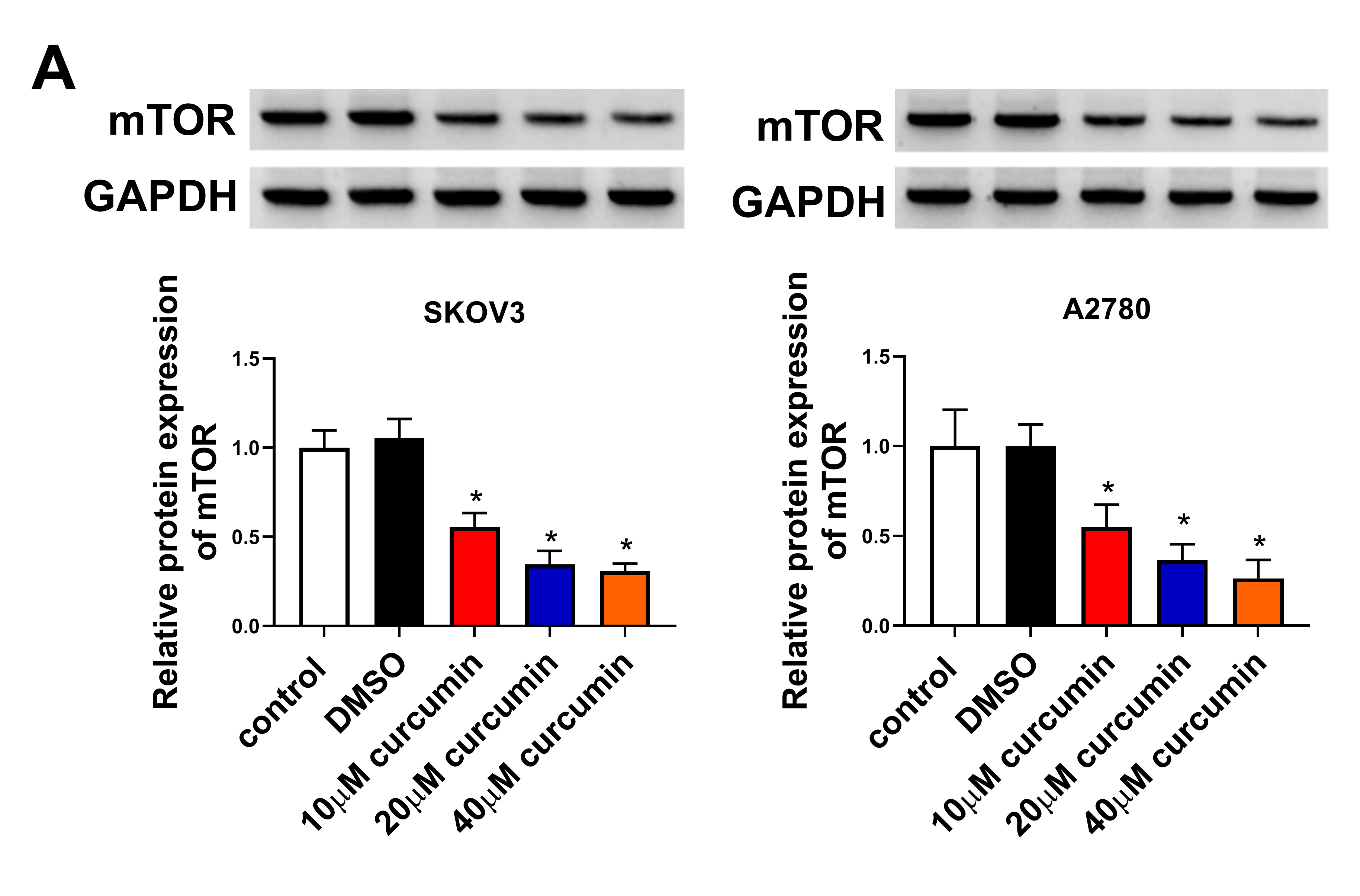

Supplement: Supplementary file 4 — Additional file 4. [file 13048_2021_916_MOESM4_ESM.tif]
